# *Akkermansia muciniphila* and osteoporosis: emerging role of gut microbiota in skeletal homeostasis

**DOI:** 10.3389/fmicb.2025.1665101

**Published:** 2025-09-15

**Authors:** Yanlong Gong, Xin Ma, Jiumei Huang, Pengwei Zhang, Yunxiang Hai, Yongjia Song, Min Song, Yuanzhen Li, Haonan Wen, Wantao Dong

**Affiliations:** ^1^Affiliated Hospital of Gansu University of Chinese Medicine, Lanzhou, China; ^2^School of Clinical Chinese Medicine, Gansu University of Chinese Medicine, Lanzhou, China

**Keywords:** osteoporosis, *Akkermansia muciniphila*, gut microbiota, skeletal health, gut-bone axis

## Abstract

Osteoporosis (OP) is a prevalent age-related skeletal disease. It is marked by compromised bone strength and higher fracture risk. Emerging evidence ties gut dysbiosis to OP development. Yet, the exact role of specific commensal bacteria remains unclear. Here, we review how *Akkermansia muciniphila (A. muciniphila)* affects bone metabolism. This mucin-degrading bacterium acts through three well-documented mechanisms: metabolite signaling, immune modulation, and gut-bone axis crosstalk. We also discuss emerging factors, such as host metabolic status, mechanical loading, and biomaterial applications. First, *A. muciniphila* produces short-chain fatty acids (SCFAs: acetate, propionate, butyrate), bile-acid metabolites, and vitamin K2. These substances boost Runx2-mediated osteoblast (OB) differentiation. They also suppress NF-κB-driven osteoclastogenesis. Second, the bacterium restores gut immune balance. It does so by expanding Foxp3^+^ regulatory T (Treg) cells and shifting macrophages toward an anti-inflammatory M2 phenotype. It also down-regulates IL-6, TNF-α, and RANKL signaling, thus limiting bone resorption. Third, via the gut-bone axis, *A. muciniphila*-derived extracellular vesicles (EVs) and miRNAs (e.g., miR-214-3p) enter the bloodstream. They strengthen intestinal barrier integrity, regulate calcium-phosphorus balance, and reduce systemic inflammation. Findings on *A. muciniphila* and bone health are conflicting. Some clinical and animal studies link higher abundance to better bone mass, with depletion worsening OP. Others, however, report negative correlations between *A. muciniphila* levels and bone mineral density (BMD) in separate cohorts. Most data come from pre-clinical models. Long-term human studies are scarce, and no clear causal links have been established. Future research should focus on randomized controlled trials. These trials need to define strain-specific effects, optimal doses, and safety profiles. The goal is to resolve these inconsistencies and turn *A. muciniphila*-based approaches into precise therapies for preventing and treating OP.

## 1 Introduction

Osteoporosis is a skeletal disorder characterized by reduced bone mass and micro-architectural deterioration, affecting approximately 18 % of adults worldwide and accounting for tens of billions of dollars in fracture-related health costs (Cosman et al., 2024). Current anti-resorptive and anabolic agents can diminish bone loss but fail to fully restore bone quality, and long-term use is limited by adverse effects (Fuggle et al., 2024). Consequently, novel therapeutic avenues are urgently needed.

With aging, the gut microbiota (GM) undergoes a marked loss of diversity and functional capacity, leading to impaired barrier integrity, altered short-chain fatty-acid (SCFA) production, and chronic low-grade inflammation (Martino et al., 2022; Purse et al., 2025). These dysbiotic changes overlap with the pathophysiological hallmarks of OP, suggesting that age-related microbial disturbances may be an under-appreciated driver of bone loss.

The gut-bone axis denotes a bidirectional communication network whereby intestinal microbes influence skeletal remodeling and, reciprocally, bone metabolism can shape the gut environment (Hansen and Frost, 2022). While broad dysbiosis has been linked to reduced BMD, the contribution of specific keystone taxa–notably *A. muciniphila*–remains poorly defined.

*A. muciniphila* is increasingly recognized as a health-promoting commensal in metabolic and inflammatory disorders (Cani et al., 2024), yet its mechanistic role in bone homeostasis and OP has not been systematically examined. We collate and critically evaluate the emerging evidence that connects *A. muciniphila* to skeletal health, dissect the underlying molecular pathways (microbial metabolites, immune modulation, gut-bone signaling, and host-microbe interactions), and assess the translational potential of targeting this organism for OP prevention or therapy.

In the following sections we (I) summarize the distinctive biology of *A. muciniphila* (Section 2), (ii) examine the association between *A. muciniphila* and OP (Section 3), (iii) review intervention strategies (Section 4), (iv) discuss therapeutic benefits an safety considerations (Section 5), and (v) outline future research directions (Section 6).

## 2 Biological characteristics of *A. muciniphila* and its role in host health

### 2.1 Structural and metabolic features

As a member of the Verrucomicrobia phylum, *A. muciniphila* primarily colonizes the colonic segment, with secondary niches in the small intestine (jejunum and ileum) and cecum of mammals (Geerlings et al., 2018). These bacteria flourish in the intestinal mucus layer, utilizing host-secreted mucin as their primary nutrient source. During host fasting, it strategically stores mucin degradation products to maintain GM homeostasis (Ioannou et al., 2025). While it can metabolize limited oligosaccharides (e.g., fucose), this process occurs with significantly lower efficiency compared to mucin catabolism. Notably, *A. muciniphila* demonstrates metabolic plasticity in processing glucosamine derivatives. Its efficient utilization of N-acetylglucosamine not only enhances bacterial proliferation but also improves intestinal barrier function and reduces chronic low-grade inflammation (Nie et al., 2024).

Being a resident component of the human GM, *A. muciniphila* is categorized as a probiotic. By degrading mucopolysaccharides, *A. muciniphila* produces SCFAs, including acetate, propionate, and butyrate, which contribute to maintaining intestinal barrier integrity and metabolic homeostasis. Besides, *A. muciniphila* positively regulates the thickness of the intestinal mucus layer and enhances barrier integrity through the competitive exclusion of pathogens (Zhang et al., 2024c). Studies have shown that *A. muciniphila* significantly strengthens gut barrier integrity, leading to a marked decrease in plasma lipopolysaccharide (LPS) levels (Lin et al., 2024). This LPS modulation critically affects redox homeostasis, as LPS can increase the generation of reactive oxygen species (ROS), including lipid peroxides, superoxide radicals, and nitric oxide derivatives, while also inducing ferroptosis in intestinal epithelial cells (Wang and Hu, 2023). In addition, the bacterium also exhibits immunomodulatory properties through its membrane components, though the exact mechanisms require further elucidation (Xie et al., 2024). In addition, Current studies indicate that *A. muciniphila* plays a significant role in modulating obesity, inhibiting inflammation, modulating immunity, and addressing various metabolic disorders (Zeng et al., 2023; Zhu et al., 2023).

### 2.2 Host interaction and health implications

**FIGURE 1:**
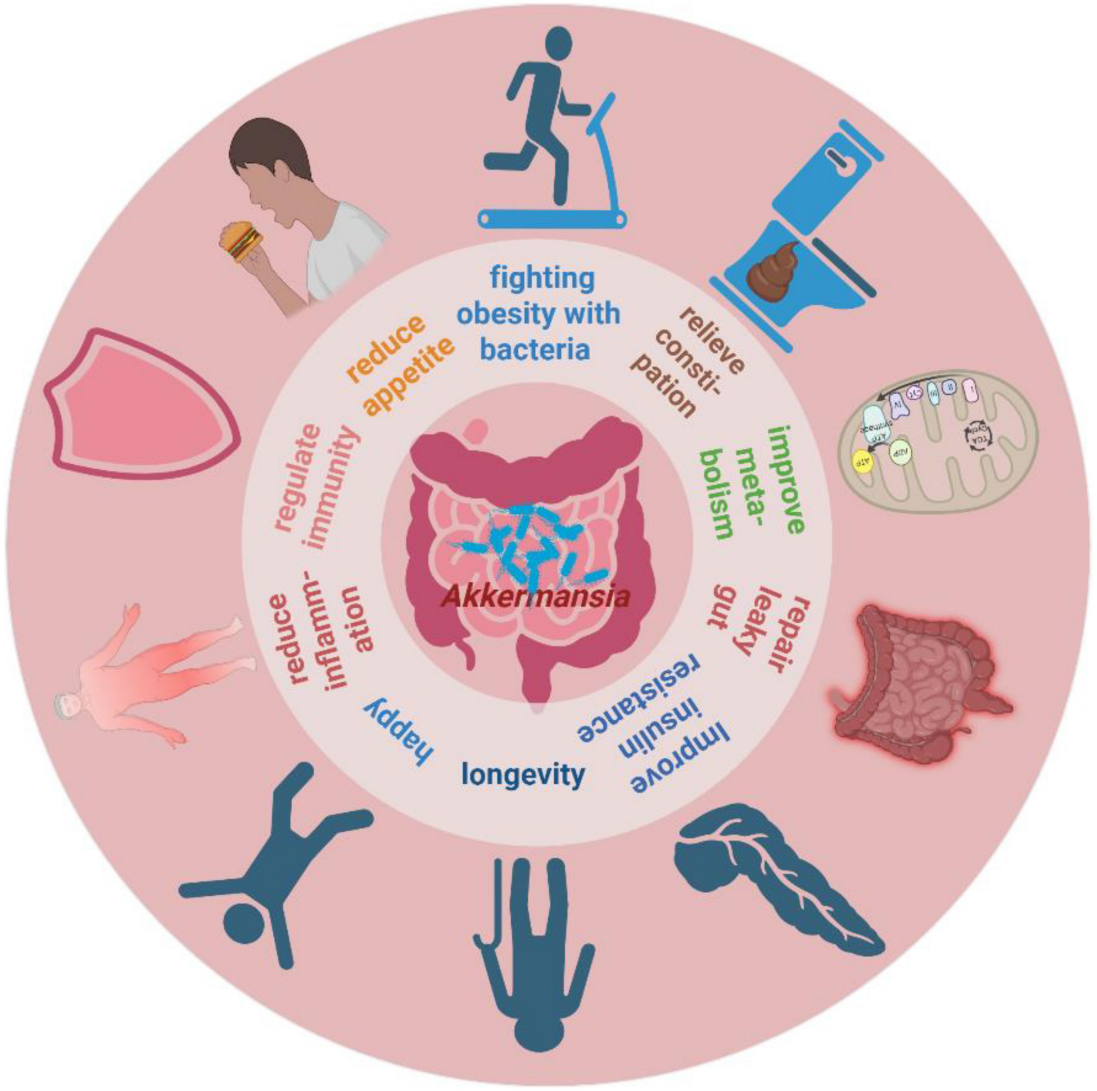
The potential roles of *A. muciniphila* for host health. Created in BioRender. Xin, M. (2025), https://biorender.com/l5m3y5r.

Current studies indicate that *A. muciniphila* abundance is associated with various metabolism-related diseases, such as obesity, diabetes, cardiovascular disorders, bone metabolism disorders, neurodegenerative diseases, immune and inflammatory diseases, and chronic kidney disease (Zeng et al., 2023). Moreover, *A. muciniphila* has been identified as a “longevity-promoting bacterium” due to its ability to slow aging and extend lifespan by modulating microbial homeostasis, inflammation, and immune metabolism (Kadyan et al., 2025). Additionally, *A. muciniphila* enhances host health by increasing resistance to pathogen colonization (Spragge et al., 2023) and interrupting pathogen transmission (Zhang et al., 2024b). These findings underscore the multifaceted contributions of *A. muciniphila* to maintaining overall health and homeostasis (Wang et al., 2017; Sun et al., 2024; Aja et al., 2025). See Figure 1 for an overview of these contributions.

*A. muciniphila* enhances gut barrier function and reduces systemic inflammation by competitively excluding pathogens and reinforcing the intestinal mucus layer. Excessive mucus production can alter the GM structure and increase the abundance of mucus-preferring bacteria such as *A. muciniphila*, which helps prevent chemically and microbiologically induced intestinal inflammation (Naama et al., 2023). Given its broad influence on metabolic and inflammatory pathways, *A. muciniphila* holds promise for clinical applications in metabolic and age-related disorders.

## 3 The association between *A. muciniphila* and OP

**FIGURE 2:**
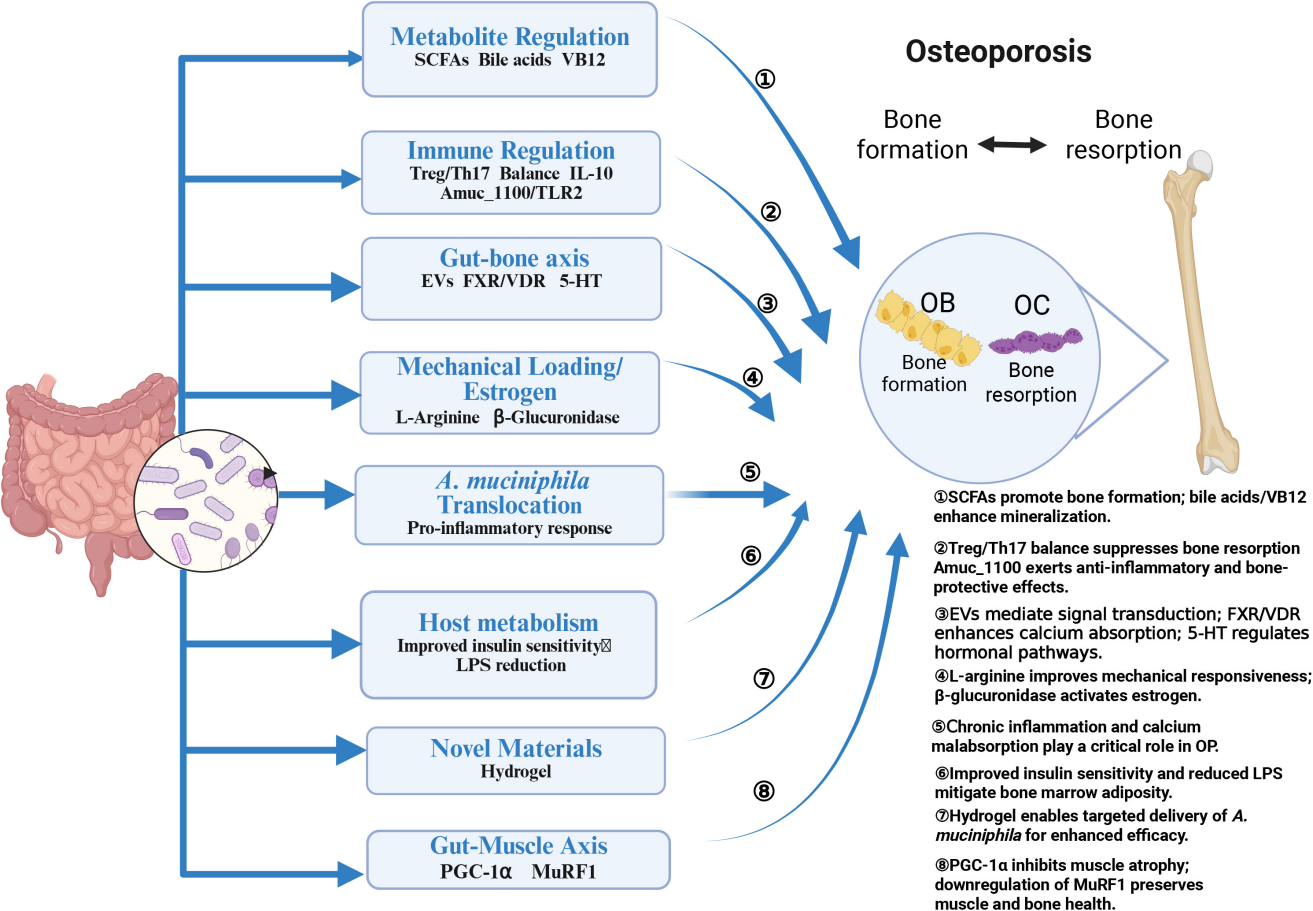
Effects of *A. muciniphila* on OP. Created in BioRender. Xin, M. (2025), https://biorender.com/fxpn7c9.

Over the past few decades, researchers have extensively investigated GM composition in various diseases, including inflammatory bowel disease (Lavelle and Sokol, 2020), type 2 diabetes (Wu et al., 2020), aging (O’Toole and Jeffery, 2015), obesity (Kahleova et al., 2020), and OP (Chen C. et al., 2024). Numerous studies have shown a significant correlation between gut *A. muciniphila* abundance and the pathogenesis of these conditions. Recent findings indicate that GM composition undergoes significant changes with advancing age (Jeffery et al., 2016), marked by reduced diversity, functional alterations, and significant inter-individual variability (Claesson et al., 2012; Van Hul et al., 2024). Primary OP, a disease closely linked to aging, diabetes, and obesity, is thought to be influenced by these microbiota-related shifts (Li et al., 2020). Against this backdrop, the specific effects of *A. muciniphila* on OP, as visualized in Figure 2, warrant detailed discussion.

### 3.1 Microbial metabolites and osteocyte function

*A. muciniphila*, a key member of next-generation probiotics (NGPs) (Abbasi et al., 2024), produces a variety of bioactive compounds, including SCFAs such as acetate, propionate, butyrate, succinate, and sulfate; 1,2-propanediol; indole-3-acetic acid (3-IAA); secondary bile acids; γ-glutamyl amino acids; and tryptophan-derived signaling molecules. This commensal bacterium also synthesizes structurally unique components, including the branched phospholipid species a15:0-i15:0 phosphatidylethanolamine, the outer membrane protein Amuc_1100, and EVs (collectively termed *A. muciniphila*-derived EVs, or AMEVs). These metabolites and cellular components collectively regulate intestinal immunometabolic homeostasis by modulating host-microbe interactions. They play crucial roles in maintaining gut barrier integrity, regulating energy balance, and modulating mucosal immune responses.

SCFAs, primarily produced through the gut microbial fermentation of dietary fibers and mainly consisting of acetate, propionate, and butyrate (Mann et al., 2024), promote skeletal health by multitargeted regulation of bone metabolism. These compounds modulate osteocyte function, coordinate immune responses, regulate endocrine environments, and enhance nutrient absorption efficiency (Zhang et al., 2024d). Specifically, butyrate produced by *A. muciniphila* stimulates OB differentiation by inhibiting histone deacetylase (HDAC) (Hays et al., 2024). This metabolite upregulates Runx2 and Osterix expression in bone marrow mesenchymal stem cells (BMSCs), thereby promoting osteogenic commitment while simultaneously inhibiting adipogenesis through the downregulation of peroxisome proliferator-activated receptor gamma (PPARγ) (Hu et al., 2014).

Propionate generated by *A. muciniphila* activates G protein-coupled receptor 43 (GPR43), directing BMSCs toward OB differentiation rather than adipocyte formation (Al-Lahham et al., 2010). This metabolite upregulates osteogenic transcription factors Runx2 and osteocalcin (OC) via the AMPK/mTOR pathway, while also increasing bone formation markers alkaline phosphatase (ALP) and osteoprotegerin (OPG) (Thangaraju et al., 2009). Additionally, propionate directly suppresses osteoclast activity by reducing tartrate-resistant acid phosphatase 5b (TRACP-5b) levels and enhancing OC secretion, confirming its dual role in bone remodeling (Li Z.-X. et al., 2024).

Bile acids are crucial for metabolic regulation and inflammatory modulation. *A. muciniphila* enhances intestinal calcium absorption by remodeling the host bile acid pool, increasing the ratio of secondary bile acids to lithocholic acid (LCA). This process activates intestinal epithelial cells via the Farnesoid X Receptor (FXR), upregulating the calcium transporter TRPV6 and promoting bone mineralization (Nie et al., 2024). A groundbreaking study by Bárcena et al. (2019) showed that progeroid mice exhibited enriched “secondary bile acid biosynthesis” pathways following *A. muciniphila* intervention. The treatment significantly increased intestinal trefoil factor Tff3, restored depleted metabolites (including arabinose, ribose, inosine, and ether phospholipid PCae [18:0]), alleviated pathological phenotypes (such as osteolysis, OP, and systemic lipoatrophy), and extended lifespan in progeroid models (Bárcena et al., 2019). Mechanistically, LCA further enhanced bone mineralization through FXR-mediated upregulation of the Vitamin D Receptor (Lyu et al., 2024).

Notably, the capacity for vitamin B12 (VB12) biosynthesis is a significant metabolic divergence among *A. muciniphila* subspecies. Approximately 30% of *A. muciniphila* isolates possess complete operons for VB12-dependent propionate synthesis, while specific strains also contribute to vitamin K2 (VK2) production (Yan et al., 2022). As an essential coenzyme for OC carboxylation, VK2 directly regulates hydroxyapatite crystallization during bone matrix maturation.

Metabolites derived from *A. muciniphila* act as crucial regulators of osteocyte differentiation and function. These compounds promote the proliferation and differentiation of osteoprogenitor cells, supporting their development into functional osteocytes. In addition, they enhance OB activity, driving bone matrix formation and mineralization, and thereby boosting skeletal growth and repair. In contrast, metabolites from *A. muciniphila* inhibit osteoclast formation and activity, effectively reducing bone resorption and slowing the progression of OP. These regulatory effects are mediated through intricate signaling mechanisms involving key transcription factors and signaling molecules that collectively modulate gene expression and cellular behavior in bone tissues.

### 3.2 Immunomodulation and inflammation regulation

The gastrointestinal tract hosts 70%–80% of the body’s immune cells, including T cells, B cells, and NK cells, which are concentrated in the intestinal mucosa and serve as the primary defense against pathogenic invasion (Hanna et al., 2023). *A. muciniphila* plays a critical role in modulating immune functions through multiple pathways: (a) Phospholipid-Mediated Barrier Enhancement: Phospholipids derived from *A. muciniphila*, such as a15:0-i15:0 phosphatidylethanolamine, stimulate mucin production, thereby reinforcing intestinal barrier integrity. This reduces pathogen translocation and modulates immune responses (Bae et al., 2022). These phospholipids also regulate dendritic cell activation and subsequent T/B lymphocyte functionality (Luo et al., 2022). (b) Cytokine Network Regulation: *A. muciniphila* modulates inflammatory responses by regulating the secretion of cytokines such as TNF-α and IL-6 by immune cells (Chen et al., 2023). (c) Microbial Consortium Synergy: *A. muciniphila* forms a homeostatic alliance with commensal bacteria like Clostridiales and Ruminococcaceae. Together, they prevent intestinal hyperpermeability and systemic immune dysregulation (Routy et al., 2018).

The “*A. muciniphila*-Treg-Bone Immunology Axis” clarifies the skeletal regulatory mechanisms of *A. muciniphila*. Enrichment of *A. muciniphila* increases the population of Foxp3^+^ regulatory Treg cells in the intestinal lamina propria (Liu et al., 2022). These Tregs suppress osteoclastogenesis by: (a) Secretion of IL-10 to inhibit receptor activator of nuclear factor kappa-B ligand (RANKL) activation in osteoclast precursors. (b) Upregulation of OPG expression to counteract bone resorption. (c) Germ-free murine models have shown that *A. muciniphila* deficiency exacerbates the activation of RORγt + T helper 17 (Th17) cells, driving IL-17A-mediated osteoclast differentiation (Abraham and Medzhitov, 2011; Peng et al., 2024). Supplementation with *A. muciniphila* restores the balance between Tregs and Th17 cells and attenuates RANKL/RANK signaling, effectively preserving bone mass.

Depletion of *A. muciniphila* induces a shift in intestinal macrophages toward the M1 pro-inflammatory phenotype, characterized by increased secretion of IL-1β and TNF-α. These cytokines activate the NF-κB signaling pathway, accelerating osteoclastogenesis. Conversely, *A. muciniphila* supplementation reverses this polarization, promoting the M2 anti-inflammatory phenotype (ARG1+) while suppressing NF-κB activation (Grivennikov, 2013; Zhang X. et al., 2025). This immunomodulatory effect reduces the release of TNF-α and IL-1β, inhibits the differentiation of osteoclast precursor cells, and improves the bone microenvironment, ultimately restoring bone metabolic homeostasis.

*A. muciniphila* mediates anti-inflammatory and osteoprotective effects through the production of SCFAs, which enhance the differentiation of Treg cells. This suppresses excessive inflammation and reduces the secretion of IL-6 and TNF-α, indirectly attenuating osteoclast activity. Unlike other probiotics, *A. muciniphila* colonizes the intestinal mucosal layer, where its outer membrane protein Amuc_1100 activates TLR2, strengthening epithelial tight junctions and mucosal barrier function (Plovier et al., 2017). This mechanism inhibits the leakage of bacterial antigens into epithelial tissues, reduces systemic inflammation, and limits the translocation of LPS into the circulation, thereby suppressing bone marrow adipogenesis (Liu et al., 2021). Experimental studies show that both *A. muciniphila* and Amuc_1100 significantly reduce *Porphyromonas* gingivalis-induced inflammatory responses by decreasing TNF-α and IL-6 production while promoting the polarization of macrophages toward the anti-inflammatory M2 phenotype. This intervention reduces alveolar bone loss by 30%–50% in periodontitis models, confirming its osteoprotective capacity (Mulhall et al., 2020). In murine fracture models, treatment with *A. muciniphila* restores intestinal barrier integrity disrupted by dextran sulfate sodium (DSS), alleviates local inflammation, and induces the formation of platelet-derived growth factor BB-positive (PDGF-B+) osteoclasts and H-type vessels at bone injury sites, ultimately accelerating fracture healing (Liu et al., 2020).

Osteoporosis pathogenesis involves gut dysbiosis, chronic inflammation, immune dysfunction, and stress responses–all of which can be targeted through GM modulation for OP management. In OP patients, the compromised intestinal barrier integrity allows bacterial translocation. This enables gut-derived bacteria and toxins to spread via systemic circulation to distant organs, including the lungs, kidneys, brain, and skeletal tissues (D’Amelio and Sassi, 2018). Such microbial translocation triggers localized and systemic inflammation, immune dysregulation, and multi-organ dysfunction, thereby worsening disease progression (Camilleri, 2019; Su et al., 2024). As a keystone species for mucosal barrier maintenance, *A. muciniphila* strengthens intestinal defenses through three complementary mechanisms: Stimulating mucus secretion to reinforce the physical barrier. (a) Producing β-N-acetylhexosaminidase to regulate tight junction proteins. (b) Generating SCFAs to stabilize the mucosal microenvironment (Zheng et al., 2023). (c) While direct evidence linking *A. muciniphila* translocation to OP prevention is still lacking, its therapeutic potential may involve site-specific microbial interactions. Further investigation through translocation-focused studies is warranted.

*A. muciniphila* indirectly regulates bone metabolism through immunoregulatory mechanisms, primarily by controlling inflammatory responses and immune cell activity to maintain skeletal homeostasis. This is achieved through enhanced intestinal barrier function, stabilized immune equilibrium, balanced cytokine production, and coordinated interactions between immune cells and bone cells. Clinical studies have established a positive correlation between *A. muciniphila* abundance and bone mass (Chen C. et al., 2024), indicating that its immunomodulatory properties may serve as a potential therapeutic target for reducing inflammatory bone loss.

### 3.3 Gut-bone axis and systemic communication

**FIGURE 3:**
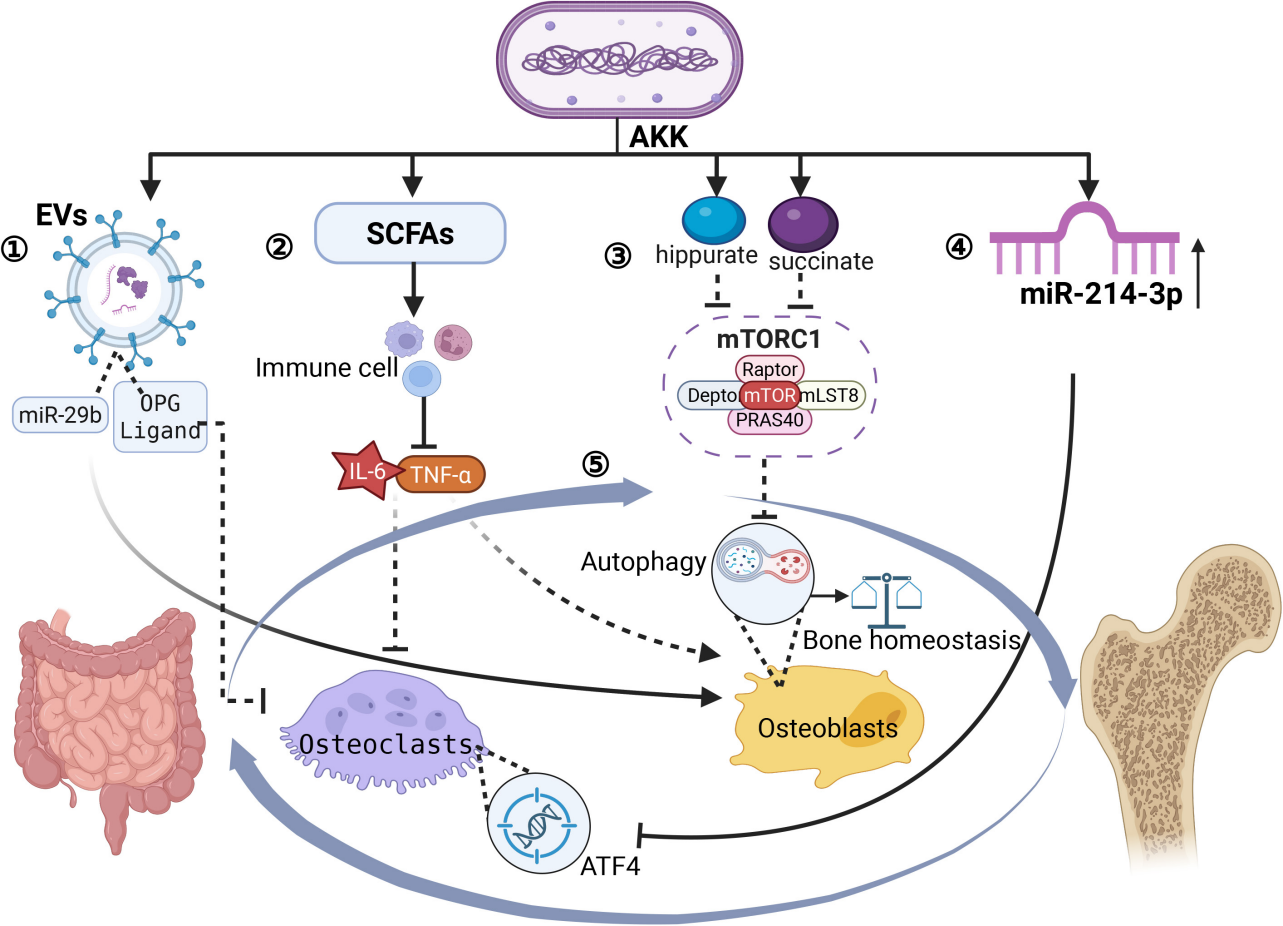
Gut-bone axis governed by *A. muciniphila*. Created in BioRender. Xin, M. (2025), https://biorender.com/fxpn7c9. ➀ *A. muciniphila* releases EVs → blood; EV cargo: miR-29b **+** OPG-ligand **+** propionate; Uptake by OBs/osteoclasts; miR-29b → OB proliferation; OPG-ligand → blocks RANKL-RANK →↓ osteoclast activity →↑ bone density & strength. ➁ SCFAs (propionate, acetate) →↓ gut inflammation → systemic anti-inflammation → enhanced mineralization. ➂ Succinate & hippurate → enter bloodstream → activate mTORC1 → modulate OB autophagy→ enhanced mineralization. ➃ *A. muciniphila* ↑serum miR-214-3p→suppress ATF4 in osteoclasts →osteoclast activity↓→reduced bone loss →protection against OP. ➄ A reciprocal interaction exists between the gut and skeletal systems.

The “cross-talking” mechanism of the gut-bone axis, mediated by *A. muciniphila*, involves a complex multi-organ regulatory network that integrates microbial metabolism, immunomodulation, and direct signaling pathways. This process includes extracellular vesicle (EV)-mediated interorgan communication, systemic immune regulation, the endocrine effects of metabolites, microbiota-driven calcium-phosphorus homeostasis, miRNA-based cross-tissue regulation, and neuroendocrine signaling. A schematic diagram synthesizing the main mechanisms is provided in Figure 3 for clarity.

Liu et al. (2021) discovered a novel gut-bone communication pathway through bacterial-derived functional EVs. They demonstrated that EVs produced by *A. muciniphila* in children can traverse intestinal barriers to reach bone tissues, directly modulating osteocyte activity. These EVs transport miR-29b, OPG ligands, and metabolites, which are internalized by OBs and osteoclasts to regulate their proliferation and differentiation. Animal studies have shown that intravenous administration of these pediatric GM-derived EVs significantly enhances bone density and mechanical strength. *A. muciniphila* may enhance this process by improving intestinal barrier integrity, thereby facilitating systemic EV distribution. Meanwhile, its metabolites, such as propionate, synergize with EV cargo to activate bone formation pathways (He et al., 2023, Geng et al., 2025).

The pathogenesis of OP is closely associated with chronic inflammation. SCFAs produced by *A. muciniphila* modulate intestinal immune cell function, reduce systemic inflammation, and indirectly regulate bone metabolism (Rodrigues et al., 2022). Microbial metabolites like serum succinate and hippurate are transported via enterocytes into the circulatory system. There, they activate the mechanistic target of rapamycin complex 1 (mTORC1), thereby controlling OB autophagy and maintaining skeletal homeostasis (Zhang et al., 2023). Additionally, *A. muciniphila* optimizes intestinal barrier function, enhancing calcium and phosphorus absorption through the microbiota-gut-bone axis. This process promotes mineral ionization and improves the efficiency of bone matrix mineralization (Ioannou et al., 2025).

*A. muciniphila* regulates bone resorption by increasing host serum levels of miR-214-3p, which directly suppresses the expression of activating transcription factor 4 (ATF4) in osteoclasts. A deficiency in *A. muciniphila* reduces miR-214-3p levels, resulting in enhanced osteoclast activity and accelerated bone loss (Li et al., 2016). Furthermore, acetate derived from *A. muciniphila* activates intestinal enterochromaffin cells to secrete serotonin (5-HT). This serotonin crosses the blood-brain barrier and regulates the hypothalamic secretion of parathyroid hormone (PTH) and calcitonin (CT), thereby coordinating bone metabolism through the vagus nerve-gut-bone axis (Li R. et al., 2024).

In addition to its effects on bone health, *A. muciniphila* also plays a significant role in modulating skeletal muscle metabolism and function through the gut-muscle axis. The composition and diversity of GM significantly regulate skeletal muscle metabolism and function, as seen in catabolic conditions like sarcopenia and cachexia (Bischoff, 2016; Giron et al., 2022), as well as in anabolic states associated with athletic performance (Munukka et al., 2018). This bidirectional interaction forms the basis of the “gut-muscle axis” hypothesis, which suggests that microbial communities directly or indirectly modulate muscle mass homeostasis (Gielen et al., 2023). From an embryological perspective, the musculoskeletal system originates from common mesodermal precursors, with muscle and bone sharing a mesenchymal lineage commitment (Loh et al., 2016). Clinically, sarcopenia and OP exhibit age-related synergistic effects due to shared mechanical, cytokine-mediated, and signaling pathway dysregulations.

Hong et al. (2024) identified synergistic effects between the administration of *Eubacterium nodatum* and *Eubacterium ventriosum*, which increased *A. muciniphila* abundance, activated osteoanabolic pathways, and inhibited muscle proteolysis. This is likely mediated by SCFAs derived from *A. muciniphila* that epigenetically regulate lineage commitment in mesenchymal precursors. Based on the existing evidence regarding the multifaceted roles of *A. muciniphila* in modulating host metabolism and immune responses, we propose the following hypothesis: *A. muciniphila* promotes the increase of skeletal muscle mass through the mechanisms of SCFAs production and immune regulation, enhances mechanical loading, and thereby indirectly improves skeletal adaptability.

### 3.4 Host-microbe interactions and lifestyle factors

#### 3.4.1 Host metabolic status significantly impacts *A. muciniphila* abundance and functionality

A high-fat diet (HFD) reduces the thickness of the intestinal mucus layer, thereby depleting the ecological niche of *A. muciniphila*. Experimental evidence shows that mice fed a diet containing 60% fat for 8 weeks exhibit a 100-fold decrease in *A. muciniphila* colonization (Everard et al., 2013). However, supplementing with *A. muciniphila* restores claudin-1 expression at tight junctions, reduces circulating LPS levels, and suppresses the production of IL-6 and TNF-α in the bone marrow. These changes collectively enhance BMD. In clinical settings, obese individuals have markedly lower levels of *A. muciniphila* compared to healthy individuals. This depletion worsens metabolic comorbidities such as diabetes (Thingholm et al., 2019; Yan et al., 2021). Administering pasteurized *A. muciniphila* improves insulin sensitivity by approximately 30%, reduces hyperinsulinemia and hypercholesterolemia, and lowers obesity-related cardiometabolic risks, all without adverse effects (Depommier et al., 2019).

The low abundance of *A. muciniphila* has now been confirmed to be associated with an increased risk of obesity and is significantly correlated with OP (Morshed et al., 2024). The pathogenesis of OP involves chronic inflammation driven by obesity, which impairs *A. muciniphila* activity. This impairment compromises intestinal barrier integrity, allowing LPS to translocate into the circulation. Consequently, bone marrow adipogenesis is promoted at the expense of osteogenesis. Clinical interventions show that a 12-month supplementation with viable *A. muciniphila*, combined with calcium and vitamin D3, significantly increases lumbar spine bone density in OP patients. It also raises serum levels of procollagen type I N-terminal propeptide (PINP), a marker of bone formation, and reduces levels of C-terminal telopeptide of type I collagen (CTX-I), a marker of bone resorption. This intervention demonstrates excellent gastrointestinal tolerability (Rubanov et al., 2019; Zhu et al., 2025).

#### 3.4.2 Mechanical loading, estrogen, and bone health

Mechanical loading is essential for maintaining skeletal homeostasis and resisting OP. Wang et al. (2024) demonstrated that GM depletion significantly impairs bone mechanoadaptation to mechanical stimuli. Specifically, the proteolytic cleavage of Amuc_1100 by intestinal enzymes releases L-arginine (He et al., 2024), a critical mediator of skeletal mechanoresponsiveness. In aged and ovariectomized murine models, L-arginine supplementation activates a nitric oxide-calcium positive feedback loop within osteocytes, thereby enhancing bone’s adaptive response to mechanical loading.

Postmenopausal osteoporosis (PMOP), primarily driven by estrogen deficiency, exemplifies the hormonal regulation of bone metabolism. Estrogen directly suppresses the production of osteoclastogenic cytokines by hematopoietic stem cells, inhibits the proliferation of monocyte-derived osteoclast precursors, and induces apoptosis in mature osteoclasts, thereby inhibiting bone resorption through multiple mechanisms (Yao et al., 2025). The concurrent decline in androgen levels increases skeletal sensitivity to PTH, accelerating mineral loss (Ho et al., 2024). Recent studies have found that exercise can alter the composition of the intestinal microbiota and increase the abundance of *A. muciniphila*. This bacterium enhances intestinal barrier function and reduces systemic inflammation. Estrogen deficiency can lead to intestinal microbiota imbalance, and supplementing *A. muciniphila* can alleviate bone loss caused by estrogen deficiency by repairing the intestinal barrier, reducing endotoxemia, and inhibiting systemic inflammation (Parvaneh et al., 2015). In addition, estrogen may indirectly affect bone metabolism by regulating the metabolic products of the intestinal microbiota (Lucas et al., 2018). Furthermore, *A. muciniphila* supplementation modulated gut ecology by increasing Verrucomicrobia abundance and enhancing butyrate production. This improved oocyte quality and promoted estrogen reactivation, collectively benefiting skeletal integrity.

#### 3.4.3 Novel biomaterials and *A. muciniphila*-bone interactions

Recent advancements in biomaterial engineering have facilitated innovative applications of hydrogels, nanoemulsions, and nanodrug composites for disease prevention and treatment. Zhang et al. (2024c) developed gastrointestinal-adaptive, nutrient-self-sufficient gelatin porous microgels (*A. muciniphila*@GPMGs) to enhance the efficacy of oral *A. muciniphila* in ulcerative colitis (UC). These microgels synergistically restored intestinal barrier integrity, disrupted pro-inflammatory feedback loops, and rebalanced GM composition, achieving a 68% remission rate in murine UC models. Similarly, Zhang et al. (2024a) engineered a *Mesona chinensis* polysaccharide-polyphenol metal framework composite gel encapsulating *A. muciniphila*. This oral probiotic formulation enhanced gut ecological diversity, increased short-chain fatty acid production, and reduced dextran sulfate sodium-induced hepatotoxicity through dual barrier-immune modulation. Zu et al. (2024) further demonstrated that EVs derived from nanodrug-trained *A. muciniphila* strains (CN@Lp127s) selectively inhibited enteropathogens while promoting the growth of beneficial microbes. This offers a novel EV-based strategy for managing inflammatory bowel disease (IBD).

While no studies have yet integrated *A. muciniphila* with advanced materials for OP therapeutics, proof-of-concept platforms highlight the druggability of the gut-bone axis. An oral alginate/chitosan hydrogel microsphere system (E7-Lipo@Alg/Cs) encapsulating BMSCs and resveratrol-loaded liposomes has shown targeted BMSC delivery to bone niches. This system activates AMPK-SIRT1 signaling, reversing mitochondrial senescence and restoring osteogenic capacity in aged models (Qu et al., 2025). Propolis nanoemulsions (PNEs) with optimized gastrointestinal stability increased gut *Lactobacillus* abundance and elevated circulating L-arginine levels. These changes collectively suppressed osteoclastogenesis and enhanced OB mineralization (Zheng et al., 2024). Notably, Yu et al.’s (2024) engineered postbiotic nanosystem (B@S), containing free butyrate and *A. muciniphila*-secreted metabolites, reversed ovariectomy-induced gut dysbiosis and systemic inflammation. However, the exact microbial contributions require further investigation (Yu et al., 2024). These findings emphasize the therapeutic potential of combining microbial therapeutics with material science to precisely target OP pathophysiology through multi-scale biological interactions.

## 4 Clinical evidence and intervention strategies

Gut microbiota exhibits complex regulatory interactions with skeletal homeostasis, and *A. muciniphila* abundance can be therapeutically modulated through multifaceted interventions. Dietary strategies include consuming fruits rich in proanthocyanidins (Anhê et al., 2015); probiotic co-administration (e.g., *Bifidobacterium animalis* subsp. lactis LMG P-28149) (Alard et al., 2016); prebiotic supplementation (such as inulin and fructooligosaccharides/FOS) (Halmos et al., 2015); and following low-FODMAP diets. Pharmacological approaches involve the use of metformin (Shin et al., 2014); herbal formulations (Neyrinck et al., 2017). Lifestyle modifications such as whole-grain consumption, fiber-rich nutrition, avoiding hyperglycemic and hyperlipidemic diets and alcohol, stress reduction, and structured exercise can synergistically enhance *A. muciniphila* colonization (Zeng et al., 2023). Clinically, *A. muciniphila* has demonstrated multifunctional therapeutic benefits, including: inducing remission in the inflammatory bowel disease (Luo et al., 2022), regulating metabolic parameters encompassing body weight, adiposity, and glycemic control (Zhang X. et al., 2025), and enhancing chemotherapeutic sensitivity in gastric carcinoma management (Xu et al., 2024). Notably, a randomized double-blind trial by Depommier et al. (2019) demonstrated that pasteurized *A. muciniphila* (heat-inactivated) was more effective than viable counterparts in improving insulin resistance in obese individuals, a finding supported by preclinical models (Depommier et al., 2020). Preclinical evidence specific to OP, including details of animal models, and key outcomes related to *A. muciniphila*, is summarized in Table 1.

**TABLE 1:** The animal model research of *A. muciniphila* concerning OP.

Model type	Key findings	Conclusion	Data source
Calvarial abscess and experimental periodontitis model	In a model of calvarial infection, *A. muciniphila* decreased inflammatory cell infiltration and bone destruction. In EIP, treatment with *A. muciniphila* resulted in decreased alveolar bone loss.	*A. muciniphila* reduces Porphyromonas gingivalis-induced inflammation and periodontal bone destruction (Huck et al., 2020).	Mice
Obese murine model	NSPT and metformin improved gut microbiota and increased anti-inflammatory bacteria like *A. muciniphila*, *Lactobacillus reuteri*, and *Butyricicoccus pullicaecorum*.	NSPT and metformin enhance periodontitis and systemic bone loss treatment via gut microbiota modulation, linoleic acid metabolism recovery, and systemic inflammation amelioration (Chen et al., 2025).	Mice
Postmenopausal OP model	*A. muciniphila* enhances bone mass, reduces strength loss via EVs, and modulates bone metabolism by curbing inflammation and lipids.	Isoflavones and naringin may act on the gut-bone axis through *A. muciniphila*, potentially offering alternative postmenopausal OP treatments (Zhao et al., 2025).	Mice
Disuse-induced OP model	A decrease in the abundance of *A. muciniphila* might play a key role in the progression of disuse OP.	Each type of OP is closely, characteristically, and uniquely related to the GM and its metabolites (Qiao et al., 2024).	Rats

Emerging evidence highlights the therapeutic potential of *A. muciniphila* for obesity, OP, and sarcopenia (Deng et al., 2020; Byeon et al., 2022). Table 2 compiles evidence linking *A. muciniphila* to therapeutic effects in obesity, OP, and sarcopenia. OP patients have been found to have lower *A. muciniphila* levels than those in healthy controls (Keshavarz et al., 2021; Wang et al., 2022). Hong et al. (2024) underscored the critical interplay between host microbial genetics and therapeutic outcomes, advocating for pharmacogenomic-guided personalization of *A. muciniphila*-targeted regimens. Current research focuses on developing *A. muciniphila*-derived formulations, including live biotherapeutics, synthetic propionate analogs, and postbiotic nano-delivery systems, to enhance the prevention of OP through dietary interventions. However, optimizing these therapies will require thorough dose-response characterization and long-term safety validation across diverse populations. However, Okoro et al. (2023) identified negative correlations between *A. muciniphila* abundance and radial/tibial BMD across two independent cohorts. Notably, existing studies on the association between *A. muciniphila* and BMD are currently limited to observational correlations, and no definitive causal relationships have been established. The directionality of the association (e.g., whether *A. muciniphila* directly influences bone metabolism or reflects secondary changes in systemic homeostasis) and underlying mechanisms require further investigation through interventional studies or mechanistic experiments.

**TABLE 2:** The clinical studies on the impact of *A. muciniphila* on health.

Study type	Key findings	Conclusion	Data source
Cross-sectional study	The healthy group had higher *A. muciniphila*, non-significantly.	*A. muciniphila* load may relate to bone health, despite no significant findings (Keshavarz et al., 2021).	Patients with OP and osteopenia
Randomized controlled trial	Both groups showed reductions in body weight and glycated hemoglobin (HbA1c).	The metabolic benefit of *A. muciniphila* supplementation depends on the baseline intestinal abundance of the bacterium in each participant (Zhang X. et al., 2025).	Participants with overweight or obese type 2 diabetes
Randomized controlled trial	The HB05P group showed significantly greater improvements in left-leg extensor peak torque and peak torque normalized to body weight than the placebo group.	Pasteurized *A. muciniphila* HB05 (HB05P) is a safe postbiotic that enhances muscle strength and function in older adults, likely via up-regulation of follistatin and inhibition of myostatin (Kang et al., 2024).	Elderly adults
Meta-analysis	Obese participants exhibit a significantly lower gut-microbial diversity.	Obesity is linked to reduced gut-microbial diversity and a characteristic loss of SCFA-producers and barrier-protective bacteria, notably *A. muciniphila* (Chanda and De, 2024).	Human gut microbiota samples
Observational study	*A. muciniphila*, *B. eggerthii*, and *B. fragilis* were elevated in controls, negatively correlated with bone resorption markers, positively correlated with bone formation markers, and 25-OH-D3.	GM composition and function were widely associated with OP (Qin et al., 2021).	The elderly population with OP

## 5 Therapeutic benefits and potential risks

The translational potential of *A. muciniphila* in OP management is underscored by its multifaceted mechanisms, yet its clinical implementation necessitates careful consideration of strain-specific efficacy, host-microbe dynamics, and long-term safety. Current evidence demonstrates that *A. muciniphila* enhances skeletal homeostasis through metabolite-mediated OB promotion (e.g., SCFAs upregulating Runx2) and immunomodulatory suppression of osteoclastogenesis (via Treg expansion and M2 macrophage polarization) (Grivennikov, 2013; Zhang X. et al., 2025). Notably, pasteurized formulations such as strain HB05P have shown superior clinical translatability. Besides, the European Commission has approved pasteurized *A. muciniphila* (3.4 × 10^∧^10 CFU/day) as a novel food, highlighting its improved safety profile compared to live formulations. This approval requires explicit labeling of the bacterial inactivation status (EFSA Panel on Nutrition, Novel Foods and Food Allergens (NDA) et al., 2021).

However, therapeutic outcomes are context-dependent: while viable strains may benefit early-stage patients with intact gut barriers, their proliferation in dysbiotic conditions could exacerbate mucus layer depletion and systemic inflammation (Fernández-Tomé et al., 2021). Notably, intestinal group 3 innate lymphoid cells (ILC3s) monitor the mucosal frontier and maintain barrier integrity through IL-22 secretion (Jarade et al., 2022; Seo et al., 2022). Zhang et al. (2023) cautioned that excessive proliferation of *A. muciniphila* under ILC3-IL-22 pathway dysregulation increases succinate secretion. This could potentially activate the virulence genes of enterohemorrhagic *Escherichia coli* (EHEC), thereby triggering systemic infection. This dichotomy emphasizes the need for precision pharmacomicrobiomics–stratifying patients by baseline microbiota composition, monitoring tight junction proteins (ZO-1, occludin) and inflammatory markers (LPS, IL-6), and adjusting formulations accordingly. Critical gaps remain in understanding strain-specific interactions (e.g., vitamin K2-producing variants) (Yan et al., 2022) and synergies with conventional therapies (e.g., bisphosphonates), warranting randomized trials that standardize delivery systems (e.g., biomaterial encapsulation) and validate safety in comorbid populations.

While no direct adverse effects related to OP have been reported, the dual nature of *A. muciniphila* therapy necessitates a precision framework that integrates microbial dynamics, host genetics, and disease pathophysiology. As noted in a Cell Metabolism editorial: “Microbes are not pills–their value lies in ecosystem equilibration rather than isolated pharmacodynamics.” Developing a trilogic “microbe-host-disease” intervention system will be crucial to maximizing *A. muciniphila*’s therapeutic potential while minimizing ecological risks. To provide a clear comparison of the characteristics and potential impacts of live and pasteurized strains, the following Table 3 summarizes the differences between the two across several key aspects (Depommier et al., 2019; Ashrafian et al., 2021; Druart et al., 2021; EFSA Panel on Nutrition, Novel Foods and Food Allergens (NDA) et al., 2021).

**TABLE 3:** Comparison of key characteristics between live and pasteurized *A. muciniphila* strains.

Feature	Live strains	Pasteurized strains
Stability and safety	Live *A. muciniphila* benefits the gut, but its stability and safety may be environment-affected.	Pasteurized *A. muciniphila* has increased stability, reduced colonization and potential infection risks, thus safer.
Metabolic regulation	Improved obesity and insulin resistance, but the effect is modest.	The effect is stronger, with a marked reduction in insulin resistance and inflammatory markers.
Shelf life	Requires specific conditions (e.g., low temperature) during storage to maintain activity.	Shows better stability during storage with a 1-year shelf life.
Intestinal barrier function	No significant change in LPS levels. Significantly increased ZO-1, occludin, claudin-1, and decreased claudin-2.	Significantly reduced plasma LPS levels (*P* = 0.021). Significantly increased occludin, claudin-1, and affected ZO-1.
Effectiveness	Live *A. muciniphila* improves metabolic health, e.g., reducing diet-induced obesity, diabetes, and intestinal barrier dysfunction.	Pasteurized *A. muciniphila* retains live AKK’s benefits and even enhances them in some aspects.
Gut colonization ability	Live *A. muciniphila* can colonize the intestine.	Pasteurized *A. muciniphila* cannot colonize the intestine.

## 6 Future research directions and challenges

*A. muciniphila* has the potential to regulate bone health through various pathways, including its metabolic products such as SCFAs and vitamins, immune system modulation, “cross-talk” via the gut-bone axis, host metabolic status, mechanical loading, estrogen levels, and the gut-muscle axis. However, current research is primarily focused on basic studies, with most findings based on the indirect regulatory effects of *A. muciniphila.* There are several key challenges that must be addressed to advance *A. muciniphila*-based therapies for OP.

Firstly, the precise molecular mechanisms require further elucidation, particularly regarding strain-specific functional variations (Li et al., 2022) and gut-bone axis communication pathways. Advanced gnotobiotic models and multi-omics approaches will be critical for mechanistic insights.

Next, substantial translational gaps remain in clinical development. Interindividual variability in treatment response, stemming from differences in host genetics, baseline microbiota composition, and metabolic status, presents a major challenge (Becken et al., 2021). Furthermore, optimal dosing regimens, administration routes, and potential drug interactions with conventional OP treatments remain undefined (Sonnert et al., 2024). Well-designed clinical trials employing pharmacomicrobiomic approaches are needed to address these issues.

Furthermore, technological innovations show potential to overcome current limitations. Novel delivery systems, including biomaterial encapsulation platforms and engineered postbiotic formulations, may enhance therapeutic efficacy (Ding et al., 2025). Concurrently, standardized protocols for microbiome monitoring and analysis must be established to enable personalized treatment strategies.

Moreover, from a regulatory perspective, the development of evidence-based guidelines for microbial therapeutics is imperative. This includes establishing safety assessment frameworks, quality control standards for live biotherapeutic products, and international consensus on clinical evaluation criteria.

What’s more, Successful translation will require sustained collaboration across multiple disciplines, integrating microbiology, bone biology, clinical medicine, and regulatory science. Only through such coordinated efforts can the full therapeutic potential of *A. muciniphila* be realized for OP management. In addition to mechanistic and translational challenges, two critical aspects–personalized therapy and safety–require specific attention to advance clinical application of *A. muciniphila* in OP management.

Personalized therapy could be tailored based on an individual’s baseline gut microbiota profile (e.g., abundance of *A. muciniphila* and other commensal bacteria), genetic predispositions to bone metabolism, and metabolic biomarkers (e.g., SCFA levels, inflammatory cytokines). For instance, patients with low baseline *A. muciniphila* may benefit from targeted probiotic supplementation, while those with dysregulated immune responses might require combined interventions with anti-inflammatory agents (Shen et al., 2025). Advances in metagenomic sequencing and predictive algorithms could enable stratification of OP patients into subgroups most likely to respond to *A. muciniphila*-based therapies, thereby optimizing treatment efficacy and reducing unnecessary interventions (Chiu and Miller, 2019; Yu et al., 2023).

Safety concerns include potential systemic translocation of *A. muciniphila* in immunocompromised individuals, which may trigger opportunistic infections (Camilleri, 2019; Su et al., 2024). Additionally, long-term effects of sustained *A. muciniphila* supplementation on GM homeostasis remain understudied. Preclinical safety evaluations should include dose-dependent toxicity studies in animal models with varying immune statuses. Clinical trials should monitor adverse events (e.g., gastrointestinal disturbances, systemic inflammation) and incorporate long-term follow-up to assess microbiota stability.
